# The benefit of early acupuncture within 7 days for neurological outcomes in ischemic stroke patients after cardiac surgery

**DOI:** 10.1186/s13741-024-00470-z

**Published:** 2024-11-29

**Authors:** Chia-Hsuan Kuan, Chi-Nan Tseng, Tse-Hung Huang, Chien-Chung Yang, Yu-Sheng Chen

**Affiliations:** 1 Division of Acupuncture and Traumatology, Department of Traditional Chinese Medicine, LinKou Chang Gung Memorial Hospital, No.5, Fuxing St., Guishan Dist., Taoyuan, 333 Taiwan; 2 Division of Cardiac Surgery, Department of Thoracic and Cardiovascular Surgery, Chang Gung Memorial Hospital, Taoyuan, 333 Taiwan; 3 Vascular Surgery, Department of Molecular Medicine and Surgery, Karolinska Institute, Stockholm, Sweden; 4 School of Traditional Chinese Medicine, College of Medicine, Chang Gung University, Taoyuan, 333 Taiwan; 5 Taiwan Huangdi-Neijing Medical Practice Association (THMPA), Taoyuan, 330 Taiwan; 6 Department of Chinese Medicine, Xiamen Chang Gung Hospital, Xiamen, China

**Keywords:** Acupuncture, Aortic dissection, Cardiac surgery, Coronary artery bypass grafting, Integrative medicine, Neurological outcomes, Postoperative stroke, Stroke rehabilitation

## Abstract

**Background:**

Stroke is a critical complication of cardiac surgery that results in increased mortality and morbidity. Limited treatment options are available for patients with severe neurological deficits, such as impaired consciousness. Acupuncture is a well-known integrative management method for stroke patients. However, there are no extensive reports discussing the benefit of acupuncture in stroke patients after cardiac surgery. The aim of this study was to demonstrate the role of acupuncture in the neurological recovery of these patients and to identify the factors that provide greater benefit.

**Methods:**

This self-controlled case series utilized inpatient data from stroke patients after cardiac surgery who received acupuncture in a single center from 2013 to 2019. The primary outcomes included the Glasgow Coma Scale, muscle strength grading scale, and Barthel Index. Wilcoxon signed-rank test was used to compare the neurological differences between pre-acupuncture and post-acupuncture.

**Results:**

Fifty-one patients who met the criteria showed significant improvement of the severity of neurological impairment, including the Glasgow Coma Scale, muscle strength grading scale, and Barthel Index (*p* < 0.05). The group that underwent aortic dissection repair and the group that started acupuncture within 7 days after stroke showed greater improvement (*p* < 0.01). No adverse events were reported. Three patients with profound neurological impairment who received acupuncture intervention were described.

**Conclusions:**

Acupuncture has a potential benefit in improving neurological impairment and reducing mortality in stroke patients after cardiac surgery, especially within 7 days of the event. Further larger prospective studies with control groups are needed to provide convincing evidence.

## Background

With advancements in surgical techniques and the implementation of neuroprotective strategies in cardiac surgery, the incidence of postoperative stroke has gradually decreased (Knol et al. 2021). However, owing to the increasing number of elderly patients undergoing cardiac surgery, postoperative stroke continues to be a significant issue. According to a previous report, the in-hospital mortality of stroke patients after cardiac surgery was three times greater than that of non-stroke patients (Karunanantham et al. 2020). Impaired consciousness, muscle strength, respiratory function, and activities of daily living may further lead to prolonged hospitalization, difficulty weaning from mechanical ventilation, and a high rate of pneumonia, which imposes substantial medical and financial burdens both on families and society (Leary and Varade 2020).

Acupuncture (ACP) has long been integrated with Western medicine for stroke patients, especially in countries where it is widely practiced. Numerous studies and clinical trials have been conducted on this topic. Recent systematic reviews and meta-analyses suggest that ACP significantly improves neurological function, reduces disability, and enhances daily living activities in patients with acute stroke (Zhang et al. 2005; Zhong et al. 2024). However, most of these studies report only low to moderate certainty of the evidence. ACP has also shown certain beneficial effects on motor function, post-stroke coma, cognitive function, and swallowing function, but the low quality of the evidence underscores the need for more high-quality randomized controlled trials (RCTs) to provide clearer conclusions (Fan et al. 2024; Wu et al. 2024; Yang et al. 2016; Xu et al. 2024; Huang et al. 2022). Current research indicates that ACP may benefit stroke patients through several mechanisms: promoting neurogenesis and cell proliferation within the central nervous system, modulating blood flow in ischemic regions, reducing apoptosis in affected areas, regulating neurochemical levels, and improving long-term potentiation and memory by modulating the pathway in the hippocampus (Chavez et al. 2017). Regarding safety, reported side effects of ACP are generally minor and do not require discontinuation of treatment.

Postoperative stroke differs from ordinary stroke in etiology and pathophysiology, leading to differences in the disease process and outcomes. Traditionally, treatment options have been insufficient for stroke patients with major neurological dysfunctions, such as impaired consciousness, after cardiac surgery. Previous case reports have shown the benefit of ACP in patients with stroke after cardiac surgery, but large studies are lacking (Sheu et al. 2010; Hseuh et al. 2017).

The aim of this study was to summarize mortality and neurological differences before and after ACP in stroke patients after cardiac surgery in a medical center and to identify specific conditions under which ACP may be most beneficial, such as in patients who experienced stroke after particular surgical procedures or those who began ACP treatment early within 7 days.

## Methods

### Patient population

This retrospective self-controlled case series study was conducted at a single institution and was approved by the Human Ethics Committee of Chang Gung Medical Foundation Institutional Review Board (IRB No. 202101047B0C501). The medical records were retrospectively reviewed from a prospectively collected database of patients who received ACP treatment at LinKou Chang Gung Memorial Hospital, Taiwan, from December 2013 to December 2019. The inclusion criteria were as follows:

1. Underwent cardiovascular surgery, including aortic dissection (AD) repair, coronary artery bypass grafting (CABG), valve surgery, pericardiectomy for tumor resection, or heart transplantation
2. Diagnosis of stroke confirmed by specialized neurologists, computed tomography (CT) scans, or magnetic resonance imaging (MRI)
3. Received ACP treatment after stroke diagnosis

The exclusion criteria were as follows:

1. Diagnosis of hemorrhagic stroke
2. Diagnosis of hypoxic encephalopathy or spinal cord infarction
3. Diagnosis of stroke prior to surgery

### Assessment

All patients underwent a pretreatment assessment immediately before the first ACP session and a post-treatment assessment immediately after the final ACP session. Neurological function was evaluated via the Glasgow Coma Scale (GCS), the muscle strength grading scale (MS), and the Barthel Index (BI) before and after ACP. The GCS was used to assess the level of consciousness in stroke patients, especially in the intensive care unit, and included three components: eye opening, verbal response, and motor response. The MS evaluated weakness in each limb, with the scores summed for the analysis. The BI assessed activities of daily living to compare functional status before and after treatment and to predict prognosis (Li et al. 2019). Adverse effects were recorded during hospitalization.

### Interventions

This study employed a style of Traditional Chinese Medicine based on Huangdi’s Internal Classic (Huang Di Nei Jing). Imbalances in Qi and blood, as well as their uneven distribution, are fundamental concepts in understanding and diagnosing diseases. The condition of Qi and blood can be assessed through subtle variations in the radial pulse. ACP aims to restore the balance of Qi and blood, with subtle pulse changes observable post-treatment. In accordance with these basic concepts, acupoints in every ACP session in this study were selected based on the patient’s current clinical presentation and information obtained from a pulse examination (Yeh et al. 2021). Frequently used acupoints and detailed instructions for choosing acupoints are provided in Table 1, including their correlation with clinical presentations and specific patterns of the radial pulse (Ng et al. 2019). Four to six acupoints were selected in each session. After the skin was sterilized with 75% alcohol, stainless steel needles (0.3 × 40 mm) were inserted through the skin without manipulation. The sensation of *de qi*, defined as a feeling of soreness or heaviness around the acupoints during treatment, was not pursued in this study because most patients had impaired consciousness and were unable to provide feedback*.* The needles were retained in situ for 30 min and then removed. All patients received ACP three times a week on average until they were discharged from the hospital or passed away. All ACP procedures were performed by qualified physicians. While differences in experience might influence the overall efficacy, all practitioners were trained according to the above instructions and reached a consensus on the ACP methods and acupoint selection to minimize these differences.

**Table 1:** Frequently used acupoints, their specific pulse patterns, and the functions of the acupoints in this study

Acupoints	Related pulse	Function in TCM	Function
GV20 百會	Deficiency of the front one-third radial pulse	Redistribution of Qi and blood over the whole body	Improve consciousness and the circulation of the brain
BL2 攢竹	Deficiency of the front one-third radial pulse especially in the superficial level	Aggregate the Qi of the head and awaken the patients	
KI1 湧泉	Deficiency of the back one-third radial pulse in the deep level	Strengthen the Qi over the whole body	
LU5 尺澤	Shortness of the front one-third radial pulse	Regulate the flow of Qi above the diaphragm	Improve the function of respiratory muscle
TE17 翳風	Shortness of the front one-third radial pulse and weakness of the whole pulse	Strengthen the Qi and regulate its flow above the diaphragm	Strengthen and improve the function of respiratory muscle
LI10 手三里	Unclear boundary or low density of the front one-third radial pulse	Strengthen the Qi above the diaphragm	Strengthen the respiratory muscle
LI4 合谷	Fine and unstable pulse in superficial area	Dispel the wind and cold	Modulate the immune function and improve the function of the gastrointestinal system
ST36 足三里 ST40 豐隆	Unclear boundary or low density of the whole pulse	Supplement of Qi and blood	Alleviate fatigue, improve digestion, and enhance the immune function
ST39 下巨虛	Large, thick, or fast pulse	Decrease the inner heat	Decrease the inflammatory reaction and improve the function of digestion

### Statistical analysis

Continuous variables with a normal distribution were presented as the means ± standard deviations (SDs), whereas those with a nonnormal distribution were shown as medians with interquartile ranges (IQR). Categorical variables were presented as frequency percentages. For the analysis of baseline characteristics among groups, Fisher’s exact test was used for categorical variables, and ANOVA for continuous variables. To compare the GCS, MS, and BI scores before and after ACP, normality tests, including the Shapiro–Wilk test and Kolmogorov–Smirnov test, were first performed. While some variables were nonparametric, the Wilcoxon signed-rank test was uniformly applied. *P* < 0.05 was considered statistically significant. Statistical analyses were performed using SPSS version 20.

## Results

**Fig. 1:**
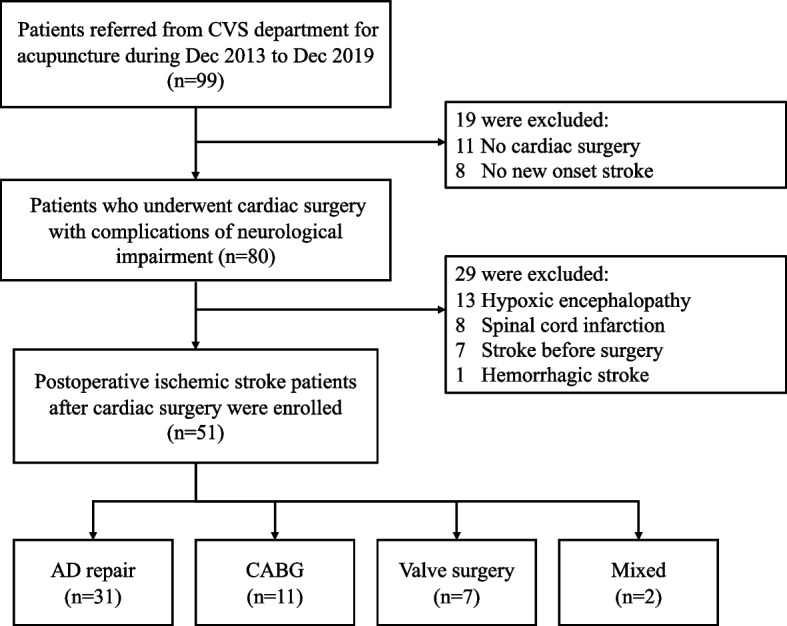
Flow chart of study recruitment. AD, aortic dissection; CABG, coronary artery bypass grafting; CVS, cardiovascular surgery

Among 99 patients referred from the cardiovascular surgery department for ACP from December 2013 to December 2019, 51 stroke patients who underwent cardiac surgery were enrolled. Forty-eight patients were excluded, and the detailed reasons are listed in Fig. 1. The basic characteristics of the enrolled patients are presented in Table 2. In terms of operative procedures, the most common operation was AD repair, accounting for 60.8% of the cases (*n* = 31), followed by CABG at 21.6% (*n* = 11) and valve surgery at 13.7% (*n* = 7). Two patients (3.9%) underwent two of the three types of surgeries. There were no significant differences in the baseline characteristics among these three groups (Table 3).

**Table 2:** Basic characteristics of the patients enrolled in the study and the patterns of ACP management

Variables	*n* = 51
**Age, mean** ± **SD**	62.53 ± 10.78
**Gender = male, *n* (%)**	29 (56.9)
**Body mass index (BMI), kg/m**^**2**^**, mean** ± **SD**	26.06 ± 4.28
**Hypertension, *n* (%)**	38 (74.5)
**Diabetes, *n* (%)**	15 (29.4)
**Initial creatinine, mean** ± **SD**	2.01 ± 1.99
**Operative procedures**	
**AD repair, *n* (%)**	31 (60.8)
Acute type A	30 (58.8)
Chronic type A	1 (2.0)
**CABG, *n* (%)**	11 (21.6)
**Valve surgery, *n* (%)**	7 (13.7)
**Mixed surgery, *n* (%)**	2 (3.9)
**ICU stay, medium (IQR)**	15 (7, 25)
**ACP frequency, median times (IQR)**	14 (9, 26)
**Interval between stroke beginning and ACP started, median days (IQR)**	4 (3, 6)
**0–7 days, *n* (%)**	40 (78.4)
> **7 days, *n* (%)**	11 (21.6)

*Abbreviations*: *ACP* acupuncture, *AD* aortic dissection, *CABG* coronary artery bypass grafting, *IQR* interquartile range, represented as (25%, 75%), *SD* standard deviation

**Table 3:** Baseline characteristics among the AD repair group, CABG group, and valve repair group before ACP

Subgroup	AD repair (*n* = 31)	CABG (*n* = 11)	Valve surgery (*n* = 7)	*P* value
**Age, mean ± SD**	61.97 ± 10.22	63.82 ± 13.62	63.14 ± 8.65	0.879
**Gender = male, *n* (%)**	21 (67.7)	5 (45.5)	2 (28.6)	0.108
**BMI, kg/m**^**2**^	26.92 ± 4.79	23.91 ± 3.34	25.56 ± 2.23	0.135
**Hypertension, *n*** **(%)**	26 (83.9)	8 (72.7)	4 (57.1)	0.219
**Diabetes, *n* (%)**	6 (19.4)	6 (54.5)	3 (42.9)	0.074
**Initial creatinine, median (IQR)**	1 (0.72, 3.69)	1.43 (0.87, 2.33)	1.37 (0.77, 1.54)	0.598
**ICU stay, median (IQR)**	15 (6, 24)	13 (10, 52)	17 (6, 36)	0.863
**Interval between stroke and ACP, median (IQR)**	4 (3, 5)	5 (3, 8)	4 (1, 5)	0.954
**ACP frequency, median (IQR)**	13 (8, 22)	20 (11, 33)	21 (9, 26)	0.929

*Abbreviations*: *ACP* acupuncture, *AD* aortic dissection, *BMI* body mass index, *CABG* coronary artery bypass grafting, *IQR* interquartile range, represented as (25%, 75%), *SD* standard deviation

^*^p < 0.05

For the patterns of ACP management, the median frequency of ACP sessions was 14 (IQR, 9, 26) (Table 2). The median time interval between the presentation of stroke and starting ACP was 4 days (IQR, 3, 6). A total of 78.4% of patients were treated with ACP within 7 days after stroke.

The overall thirty-day mortality rate following surgery was 7.8% (Table 4). Specifically, it was 6.5% for the AD repair group and 14.3% for the valve surgery group, while no deaths occurred within 30 days in the CABG group. The overall in-hospital mortality rate stood at 23.5%, with 12.9% for AD repair, 45.5% for CABG, and 28.6% for valve surgery. There were no significant differences in either 30-day or in-hospital mortality rates across the groups.

**Table 4:** Thirty-day mortality and in-hospital mortality among overall postoperative stroke patients after cardiac surgery and within each group

Mortality	*n* (%)	*p-*value
**Thirty-day mortality**	4 (7.8)	
**AD repair group**	2 (6.5)	0.112
**CABG group**	0	
**Valve group**	1 (14.3)	
**In-hospital mortality**	12 (23.5)	
**AD repair group**	4 (12.9)	0.082
**CABG group**	5 (45.5)	
**Valve group**	2 (28.6)	

*Abbreviations*: *AD* aortic dissection, *CABG* coronary artery bypass grafting

In terms of neurological outcomes, the GCS, MS, and BI all showed statistically significant differences between pre- and post-ACP (Table 5). The median differences (MD) in the GCS, MS, and BI were 1 (IQR, 0, 4; *p* < 0.001), 2 (IQR, − 1, 6; *p* = 0.002), and 5 (IQR, 0, 30; *p* < 0.001), respectively. Considering that many patients were initially intubated and on mechanical ventilation, only the motor score of the GCS, which can also reflect the level of consciousness, was independently analyzed. However, no significant improvement was observed [MD = 0 (IQR, 0, 1; *p* = 0.104)]. We used G*Power software to calculate the required sample size with an α level of 0.05 and a power of 0.8. The calculated sample size for the overall study is 36, which is smaller than the total number of participants included, indicating that the overall findings are statistically meaningful.

**Table 5:** Differences in GCS, motor score of GCS, MS, and BI before and after ACP in post-cardiac surgery stroke patients across surgical methods

		**Before ACP**	**After ACP**	**Z test**	***P* value**
		Medium (IQR)	Medium (IQR)		
**GCS**	Total	9 (7, 11)	11 (9, 15)	− 3.670	< 0.001^***^
	AD repair	9 (7, 13)	13 (9, 15)	− 3.727	< 0.001^***^
	CABG	10 (8, 12)	10 (7, 14)	− 0.476	0.634
	Valve surgery	8 (7, 10)	14 (10, 15)	− 2.220	0.026^*^
**Motor score of GCS**	Total	4 (4, 6)	6 (4, 6)	− 1.625	0.104
	AD repair	4 (4, 6)	6 (4, 6)	− 2.151	0.032^*^
	CABG	5 (4, 6)	5 (4, 6)	− 0.862	0.389
	Valve surgery	4 (4, 6)	6 (4, 6)	− 1.633	0.102
**MS**	Total	8 (5, 11)	10 (5, 15)	− 3.172	0.002^**^
	AD repair	8 (4, 10)	12 (7, 16)	− 3.413	0.001^**^
	CABG	6 (5, 9)	6 (3, 15)	− 0.307	0.759
	Valve surgery	10 (6, 13)	10 (5, 13)	− 0.106	0.916
**BI**	Total	0 (0, 0)	5 (0, 55)	− 4.461	< 0.001^***^
	AD repair	0 (0, 0)	10 (0, 55)	− 3.520	< 0.001^***^
	CABG	0 (0, 0)	0 (0, 25)	− 1.826	0.068
	Valve surgery	0 (0, 0)	20 (0, 30)	− 2.023	0.043^*^

*Abbreviations*: *ACP* acupuncture, *AD* aortic dissection, *BI* Barthel Index, *CABG* coronary artery bypass grafting, *GCS* Glasgow Coma Scale, *IQR* interquartile range, represented as (25%, 75%), *MS* muscle strength grading scale

^*^p < 0.05; ^**^p < 0.01; ^***^p < 0.001

**Fig. 2:**
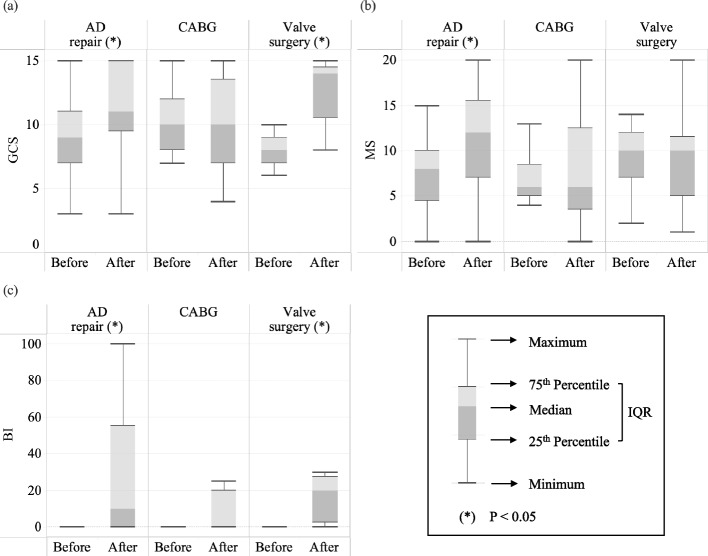
Boxplot of the patients who underwent different surgical methods, comparing **a** GCS, **b** MS, and **c** BI before and after ACP. **a** In the AD repair group, the post-ACP GCS scores of the top 75% of patients were higher than the pre-ACP median (50%). In the CABG group, the median GCS remained the same before and after ACP, but variation increased, indicating that higher scores improved while lower scores declined. In the valve surgery group, the lowest post-ACP GCS exceeded the pre-ACP median. **b** In the AD repair group, the post-ACP MS scores of top 75% of patients approached the pre-ACP median. In both the CABG and valve surgery groups, the median MS remained the same before and after ACP, but increased variation suggests that higher scores improved while lower scores declined. **c** In the AD repair group, the post-ACP BI scores of 75% of patients showed improvement. In the CABG group, BI scores of 50% of patients improved post-ACP. In the valve surgery group, 75% of post-ACP BI scores were above zero. ACP, acupuncture; BI, Barthel Index; GCS, Glasgow Coma Scale; MS, muscle strength grading scale; ^*^p < 0.05

When grouped according to different surgical procedures, there was a significant difference in the improvement in the GCS [MD = 1 (IQR, 0, 5; *p* < 0.001), motor score [MD = 0 (IQR, 0, 1; *p* = 0.032)], MS [MD = 3 (IQR, 0, 8; *p* = 0.001)], and BI [MD = 5 (IQR, 0, 35; *p* < 0.001)] before and after ACP treatment in the AD repair group (Fig. 2) (Table 5). However, the CABG group did not show a significant improvement in any of the assessments [MD of GCS = − 1 (IQR, − 3, 1; *p* = 0.634), MD of MS = 1 (IQR, − 2, 4; *p* = 0.759), MD of BI = 0 (IQR, 0, 25; *p* = 0.068)]. In the valve surgery group, the GCS and BI scores significantly improved [MD of GCS = 4 (IQR, 2, 7; *p* = 0.026), MD of MS = − 1 (IQR, − 1, 2; *p* = 0.916), MD of BI = 10 (IQR, 0, 25; *p* = 0.043)]. With only two patients in the mixed surgery group, statistical analysis using the Wilcoxon signed-rank test was not conducted. We also calculate the effect size for the subgroups; only the AD repair group met the required sample size. Therefore, the results from the CABG and valve surgery groups may not be statistically robust due to insufficient sample sizes.

**Fig. 3:**
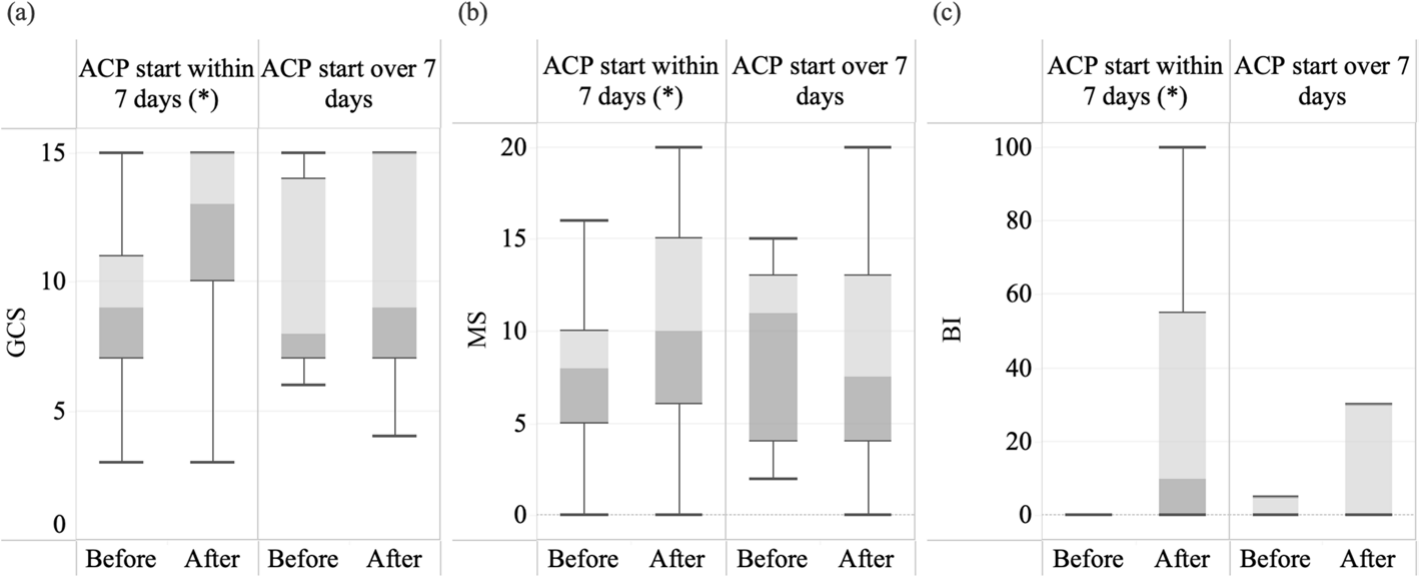
The boxplot of the patients who received ACP within 7 days and those received it over 7 days, comparing **a** GCS, **b** MS, and **c** BI. **a** In the group receiving ACP within 7 days post-stroke, the median GCS improved significantly, with the post-ACP GCS scores of the top 75% of patients exceeding the pre-ACP median. In the group receiving ACP after 7 days post-stroke, overall differences were minimal. **b** In the group starting ACP within 7 days post-stroke, the post-ACP MS scores of top 50% of patients were comparable to the pre-ACP top 25%. In the group receiving ACP after 7 days, the median MS even worsened, and the variation increased. **c** In the group receiving ACP within 7 days post-stroke, 75% of patients showed improvement in BI scores. In the group starting ACP after 7 days, improvements were minimal. ACP, acupuncture; BI, Barthel Index; GCS, Glasgow Coma Scale; MS, muscle strength grading scale; ^*^p < 0.05

Additionally, when grouped by the timing of ACP intervention, there was a significant difference in the improvement of GCS [MD = 2 (IQR, 0, 5; *p* < 0.001)], MS [MD = 2 (IQR, − 0.75, 7.5; *p* < 0.001)], and BI score [MD = 7.5 (IQR, 0, 33.75; *p* < 0.001)] before and after ACP treatment in the group that started ACP within 7 days after stroke onset (Fig. 3) (Table 6). However, in the group that received ACP after 7 days, there was no significant difference in the improvement of all assessments [MD of GCS = 0 (IQR, − 1, 1; *p* = 0.932), MD of MS = 0 (IQR, − 1, 0; *p* = 0.953), MD of BI = 0 (IQR, 0, 10; *p* = 0.109)]. No major adverse events were reported.

**Table 6:** Differences in GCS, MS, and BI before and after ACP in post-cardiac surgery stroke patients, based on ACP intervention within or beyond 7 days

	**Start time of ACP**	**Before ACP**	**After ACP**	**Z test**	***p-*value**
		Medium (IQR)	Medium (IQR)		
GCS	Within 7 days	9 (7, 11)	13 (10, 15)	− 3.835	< 0.001***
	Over 7 days	8 (7,14)	8 (7, 15)	− 0.085	0.932
MS	Within 7 days	8 (5, 10)	10 (6, 17.25)	− 3.495	< 0.001***
	Over 7 days	9 (4, 13)	7 (4, 13)	− 0.059	0.953
BI	Within 7 days	0 (0, 0)	10 (0, 55)	− 4.201	< 0.001***
	Over 7 days	0 (0, 5)	0 (0, 30)	− 1.604	0.109

*Abbreviations*: *ACP* acupuncture, *BI* Barthel Index, *GCS* Glasgow Coma Scale, *IQR* interquartile range, represented as (25%, 75%), *MS* muscle strength grading scale

^***^p < 0.001

We present two cases with positive outcomes after ACP intervention and one expired case.

### Case 1

**Fig. 4:**
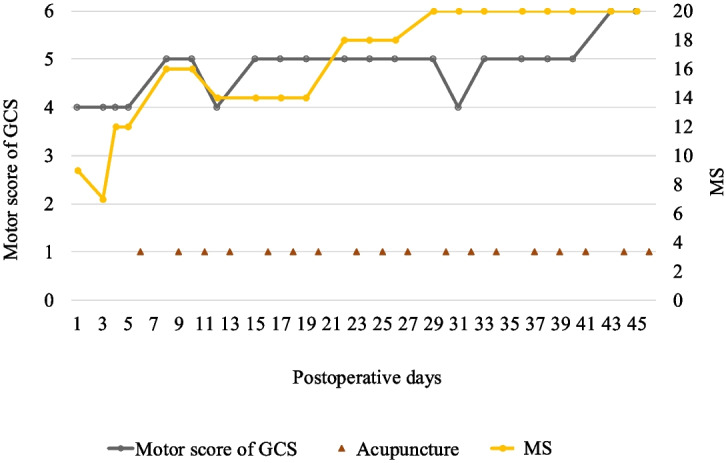
The clinical course of neurological outcomes and ACP in Case 1. ACP, acupuncture; GCS, Glasgow Coma Scale; MS, muscle strength grading scale

A 56-year-old male was brought to our emergency department because of traumatic shock after being crushed by a metal plate while working. An emergency CT revealed acute type A aortic dissection (ATAAD) and a left renal infarct. AD repair was performed. His consciousness level remained at E1VeM4 2 days after the operation. Brain CT revealed large low-density lesions in the bilateral cerebral hemispheres; therefore, massive simultaneous brain embolic stroke was suspected. His GCS score fluctuated between E1VeM4 and E4VeM4 during the initial 5 days. ACP treatment was initiated on the sixth postoperative day. His muscle strength gradually recovered to nearly normal levels by the 30th day after the operation (Fig. 4). Owing to an irritable mood and poor adherence to order, only bedside rehabilitation was suggested in the first month. After persistent ACP treatment, his level of consciousness gradually improved to E4VaM6, and he was able to cooperate by the sixth week after the operation. Therefore, the patient was transferred to the rehabilitation ward for aggressive rehabilitation programs after receiving 18 ACP sessions.

### Case 2

**Fig. 5:**
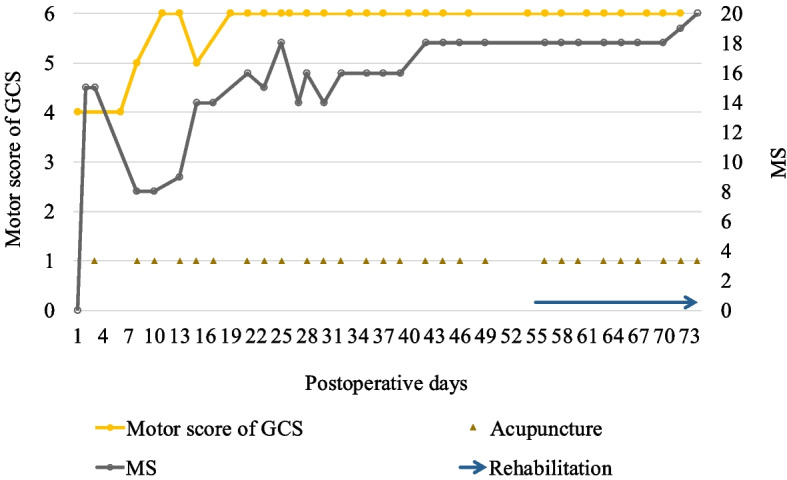
The clinical course of neurological outcomes, ACP, and rehabilitation in Case 2. ACP, acupuncture; GCS, Glasgow Coma Scale; MS, muscle strength grading scale

A 62-year-old male experienced exertional dyspnea for 3 months, accompanied by bilateral foot edema and palpitations. His medical history included hypertension, type 2 diabetes mellitus, and rheumatic heart disease with mitral valve stenosis, for which he underwent percutaneous mitral valvuloplasty 10 years ago. The dyspnea was exaggerated when the patient walked fast or upstairs. Cardiac catheterization revealed severe mitral stenosis with moderate pulmonary hypertension and impaired left ventricular systolic function, with an ejection fraction (EF) of 48.1%. The patient was then admitted to our hospital for surgical repair. However, sudden onset of left-sided weakness and slurred speech was noted 9 h after the operation. Brain CT revealed a right middle cerebral artery infarction that favored embolic stroke. ACP was initiated 2 days later. An initial consciousness status with a GCS score of E3V1M6 was noted, but several episodes of seizures occurred during the following 5 days, and repeated brain CT revealed postinfarction hemorrhage without a midline shift. His consciousness deteriorated to E1V2M4 after the seizures. The ACP was continued 3 times a week. The GCS score improved to E4V5M6 in approximately 14 days. Left limb weakness gradually improved from a MS score of 1 to a near-normal score of 5 within 2 months (Fig. 5). The patient underwent 31 ACP sessions during hospitalization.

### Case 3

A 56-year-old female presented with chest tightness with radiation to the back, and a chest CT revealed ATAAD from the aortic root to the right common iliac artery with severe intimal tears. The emergent operation of AD was performed. However, the patient did not regain consciousness after anesthesia. Follow-up CT angiography revealed left frontal infarction and right common carotid artery dissection. The operation was arranged again for dissection repair. Impaired consciousness remained with unequal pupil dilation. Subsequent brain CT revealed infarction of the left middle cerebral artery territory with hemorrhagic changes, left uncal herniation with brainstem compression, and right frontal lobe infarction. Owing to the high risk of decompression neurosurgery and the low chance of conscious regain, the neurosurgeon suggested medical treatment. After mannitol was used, the degree of brain swelling gradually improved. However, no prominent neurological improvement was noted. Supportive treatment was maintained, and ACP was started on the ninth day after the operation. After discussion with the family, they decided to enter hospice care without aggressive resuscitation. The patient died on the fourteenth day after the operation.

## Discussion

Despite the differences in underlying heart diseases and surgical methods among the patients included in this study, they all shared a common pathophysiology: interference with cerebral blood flow during cardiac surgery. This phenomenon may cause embolism or reduced cerebral blood flow, leading to stroke (Leary and Varade 2020). Embolism is caused by emboli that occur during cardiovascular surgery and during postoperative atrial fibrillation. The incidence rates of brain injury in cardiac surgery with cardiopulmonary bypass are 30–70% and 30–50% in off-pump cardiac surgery (Scolletta et al. 2015). To control for confounding factors, we excluded patients with hemorrhagic stroke, ensuring that all participants in the study had ischemic stroke. Due to the varied diagnoses for cardiac surgery, the underlying conditions of the patients differed. To further account for confounders, we conducted subgroup statistical analyses based on the different underlying cardiac conditions.

The reported incidence of postoperative stroke varies slightly among different types of cardiac surgery. It ranges from 5–16% for AD repair surgery, 0.85–4% for CABG, and 4–10% for valve surgery (Leary and Varade 2020; Scolletta et al. 2015; Dumfarth et al. 2018; Bossone et al. 2013; Hatfield et al. 2020; Yamamoto et al. 2021). Patients with postoperative stroke have a sevenfold higher operative mortality rate and more than a twofold higher long-term mortality rate compared to non-stroke patients (Hatfield et al. 2020). Stroke caused by large vessel occlusion (defined as the occlusion involving the internal carotid artery, middle cerebral artery, or basilar artery) after cardiac surgery is associated with poor survival and functional outcomes (Sultan et al. 2020).

AD is a life-threatening emergency, and the mortality rate for surgical repair is relatively high compared to other cardiac surgeries. For patients undergoing AD repair, the in-hospital mortality rate can range from 10 to 25%, depending on the severity of the dissection, the patient’s condition, and the timing of the surgery (Freitas et al. 2021; Obel et al. 2022). CABG is primarily performed to improve blood flow in patients with coronary artery disease. The operative mortality rate is generally lower than AD surgery, ranging from 1 to 3% in elective cases but can be higher in emergency or complicated cases (Stone et al. 2019; Buszman et al. 2016; Holm et al. 2020). Valve surgeries, whether for valve repair or replacement, vary depending on the type of valve involved (e.g., mitral, aortic). Mortality rates for valve surgeries are influenced by the patient’s overall health and the complexity of the procedure but are typically in the range of 1–5% (Akowuah et al. 2023; Otto et al. 2021).

The mortality rate after AD surgery is inherently high, and the occurrence of stroke further increases this risk. In previous reports, the 30-day mortality rate for stroke patients with ATAAD was approximately 21% (Fichadiya et al. 2022). In our study, there was only one case of chronic type A aortic dissection in the AD repair group. When excluding this case from the analysis, the 30-day mortality rate for stroke patients with ATAAD was 6.6%, which was relatively lower than previous findings. This suggests that ACP may have a protective effect on reducing the mortality of these patients. However, comparing neurological recovery in post-cardiac surgery stroke patients to existing studies is challenging due to the few studies available, most of which focus primarily on stroke patients without a history of cardiac surgery. These two populations are fundamentally different, making direct comparisons difficult. More research focusing on postoperative stroke patients are required. Our AD group showed better outcomes; however, this may also be related to their underlying cardiovascular condition, as this group has a lower proportion of diabetes mellitus compared to the other groups. While we cannot fully exclude the effects of the natural recovery process, our clinical experience suggests that ACP may provide potential benefits in addressing high mortality rates and neurological deficits in AD patients. Nevertheless, future investigations are essential to validate these findings.

In previous reports, the 30-day mortality rate for stroke patients undergoing CABG ranged from 14 to 19% (Roy et al. 2020; Tarakji et al. 2011). Notably, our CABG group reported no 30-day mortality. In contrast, the in-hospital mortality rate for stroke patients undergoing valve surgery was documented at 1.8 to 5.3% (Kumar et al. 2024; Stolz et al. 2024), while our results showed a significantly higher rate of 28.6%. Although positive trends were observed in the CABG and valve surgery groups, the small sample sizes in these subgroups limit the statistical robustness of our findings and warrant further investigation with larger cohorts.

While most results in the CABG and valve surgery groups were not statistically significant, the subgroup boxplot analysis still provides some insight (Fig. 2). It revealed that some patients in the CABG group deteriorated, whereas others showed improvements in GCS, MS, and BI under ACP intervention. CABG patients may have more issues with atherosclerosis or underlying diabetes mellitus, resulting in a higher risk of microvascular and macrovascular complications. This can lead to a lower recovery rate and an increased risk of subsequent morbidities (Patsouras et al. 2019). In our CABG group, one patient died from recurrent myocardial infarction, and five patients died from severe sepsis. Some evidence indicates a high risk of infection and sepsis in diabetes patients, although this remains a topic of debate (Costantini et al. 2021). Additionally, worsening cardiac function may also be a contributing factor. A hypothesis was proposed: if a patient’s condition worsens shortly after early ACP, this may suggest a greater extent of myocardial necrosis, potentially leading to subsequent heart failure. In such cases, the deteriorated GCS score may not be solely due to stroke, indicating the need for prompt scheduling of echocardiography and the early initiation of heart failure treatment pathways (Heidenreich et al. 2022). More studies will be necessary to test this hypothesis regarding the correlation between the ineffectiveness of ACP during specific periods and the progression of heart failure.

In the timeline of stroke recovery, the pathophysiological changes in the brain within the first 24 h involve cell death in the injured area. Within 7 days, an inflammatory response and scarring occur, followed by endogenous neuroplasticity and improvements in functional deficits, even until the chronic stage (over 6 months) (Bernhardt et al. 2017). Current research on the mechanisms of ACP in stroke treatment suggests that early intervention can reduce apoptosis and improve cerebral blood flow. Even in the chronic stage, ACP can still provide a benefit by promoting neurogenesis and cell proliferation (Chavez et al. 2017). However, stroke patients who start receiving ACP treatment earlier tend to have better recovery outcomes (Zhuo et al. 2021; Wang et al. 2022). If the extent of brain injury is controlled at the beginning, such as by reducing the ischemic area and the number of dead cells, the severity of neurological deficits may be lessened, increasing the chance of recovery. According to the consensus of the Stroke Recovery and Rehabilitation Roundtable in 2017 (Bernhardt et al. 2017), the acute stage of a stroke is defined as occurring within 7 days after the stroke, with inflammation as its major pathophysiology. Therefore, we also divided the patients into groups according to ACP within 7 days and over 7 days. The GCS, MS, and BI all significantly improved in the within-7-day group. However, there was no significant change observed in the over-7-day group. The small number of patients in this group may have affected the reliability of the results. In other studies, ACP has also been shown to effectively enhance neurological, cognitive, motor functions, and activities of daily living during the acute stage of stroke and the early recovery phase (Wang et al. 2022; Liu et al. 2016; Wang et al. 2020; Zhang et al. 2022). The logistical challenge of initiating ACP within 24 h post-stroke is acknowledged. Given the current situation at our hospital and most hospitals in Taiwan, initiating ACP treatment within 24 h is not widely accepted. Our findings suggest that even within 7 days, substantial benefits are achievable, underscoring the need to develop streamlined referral pathways.

Three clinical cases were added to provide valuable insights into the potential benefits and limitations of ACP in different clinical scenarios. Although *Case 1* and *Case 2* involved postoperative strokes following different surgical procedures, both patients received early ACP intervention in the acute stage. Despite initially poor GCS scores, both patients showed gradual improvement in consciousness, allowing them to participate in active rehabilitation therapy, which is crucial for their functional recovery. In fact, such cases were the impetus for conducting this study. Current strategies for treating stroke patients after cardiac surgery, such as high-dose antiplatelet medication and rehabilitation, are limited. Intravenous thrombolysis is not suitable for postoperative stroke patients because of the increasing bleeding tendency after cardiac surgery. The use of intra-arterial thrombolysis and endovascular mechanical thrombectomy with neurointerventional methods has increased in recent years (Karunanantham et al. 2020; Kashani et al. 2020). However, early detection of stroke in postoperative patients is still challenging because of anesthesia. Besides, a guidewire passing through the aortic arch to the common carotid artery and vertebral artery during the procedure may injure the surgical site of AD. There are currently no definite guidelines for these interventions in postoperative stroke patients. Among patients with impaired consciousness, sparse rehabilitation therapies are available because they are unable to obey commands. ACP should be considered in patients with no other aggressive treatment during this gap period, like *Case 1* and *Case 2*. In *Case 3*, initial worsening of neurologic deficits was noted, and ACP intervention was performed later on the ninth day after stroke. In addition to delayed intervention with ACP, recovery from stroke after cardiac surgery may also be affected by various factors, including the severity of cardiac disease, stroke type, severity of stroke, age, and comorbidities. ACP also has limitations in severe circumstances. Therefore, ongoing studies are required for identifying patients for whom ACP may provide greater benefit.

The motor score of the GCS can assess a patient’s suitability for aggressive rehabilitation. The definition of M6 is that the patient can obey commands for movement. In this study, 36.4% of patients with an initial motor response less than M6 before ACP regained M6 as calculated from the data in Table 7. A motor score less than M4 clinically indicates critical neurological deficits. When M4 was used as a cutoff point, the patients with M4 and M5 also showed statistically significant improvements in GCS [MD = 3 (IQR, 0, 6; *P* = 0.001), MS [MD = 1 (IQR, − 2, 8; *P* = 0.045)], and BI [MD = 0 (IQR, 0, 25; *P* = 0.002]. No significant improvement was found in the group with scores lower than M4, which may be related to the greater initial severity, as observed in *Case 3*, and the small number of cases. On the basis of the above results, ACP may offer potential benefits for patients with a motor score of 4 or 5, helping them to improve consciousness and to engage in rehabilitation therapy. Recent studies have also shown that ACP promotes recovery of consciousness in both clinical cases and laboratory research (Yeh et al. 2021; Tan et al. 2019).

**Table 7:** Motor response of GCS before and after ACP

*n* = 51	Motor response of GCS	
	Pre-ACU	Post-ACU
**M6,** ***n*** **(%)**	18 (35.3)	30 (58.8)
**< M6,** ***n*** **(%)**	33 (64.7))	21 (41.2)

*Abbreviations*: *ACP* acupuncture, *GCS* Glasgow coma scale

ACP has been applied in daily practice and integrated into health care in Taiwan. The National Health Insurance system in Taiwan promotes the integration of ACP in conventional care and approves its use in the treatment of neurological deficits such as new-onset stroke, spinal cord injury, and traumatic brain injury (Wei et al. 2011). As a result, an increasing number of patients have received ACP for improving neurological deficits since 2006. Over the years, we have observed the profound effects of ACP on patients with stroke following cardiac surgery, especially AD repair. Therefore, we aimed to summarize our experiences and conducted this pilot study.

This study has six limitations. First, selection bias was present, as not all cardiac surgeons were willing to integrate ACP intervention. This bias was particularly apparent in the early years, when most patients were referred by only a few surgeons. However, as the efficacy of ACP intervention becomes more evident, more surgeons may consider ACP consultation.

Second, since this was a retrospective study, the assessment of outcomes was conducted by different health care professionals, including doctors and nurses. This variation could compromise the accuracy and consistency of the results. Well-trained evaluators should be employed in future prospective studies to mitigate this issue.

Third, the study applied the GCS, MS, and BI from medical records, which may not fully reflect neurologic recovery and functional status. More wildly used assessment scales including NIHSS (Powers et al. 2019; Sato et al. 2008), Fugl-Meyer assessment (Fugl-Meyer et al. 1975; Duncan et al. 1983), and MRS (Broderick et al. 2017; Banks and Marotta 2007; Liu et al. 2020) were not be used because our patient population primarily focused on post-cardiac surgery recovery, rather than typical stroke care. Consequently, some stroke-related assessment data were incomplete or unavailable, as we were limited to existing clinical records. Additionally, SS-QOL is not widely used in clinical practice in Taiwan, where functional outcome measures like MRS and BI are more commonly employed. In future studies, we plan to incorporate these more sensitive and specific outcome measures to achieve a comprehensive evaluation of ACP’s effects.

Fourth, the outcomes were influenced by multiple confounders, including initial stroke severity, surgical methods, use of cardiopulmonary bypass (on-pump/off-pump), neuroanatomy of the stroke, hemorrhagic transformation, prolonged brain hypoxia before surgery, large vessel occlusion, and comorbidities such as sepsis, respiratory failure, and upper gastrointestinal bleeding. The lack of a control group made it difficult to eliminate these confounders, and the small sample size hindered comparative analysis of these different influencing factors. Although no control group was included, the self-controlled design helps minimize inter-patient variability, ensuring that each patient serves as their own control. We acknowledge that further prospective studies with control groups are needed to confirm these findings.

Fifth, since the study was conducted at a single center, the generalizability and representativeness of the results cannot be determined. Future studies should consider multicenter collaborations to increase the sample size and enhance the generalizability and representativeness of the findings. Sixth, this study does not provide long-term outcomes, making it difficult to assess the lasting or delayed effects of ACP and to identify any long-term side effects. Therefore, further well-designed multicenter studies with larger sample sizes, control groups, and long-term follow-up are warranted to evaluate the efficacy of ACP, the sustainability of neurologic improvement, and the predictors of outcomes after ACP in the recovery from postoperative stroke following cardiac surgery.

## Conclusions

Postoperative stroke is a major complication of cardiac surgery and is associated with increased mortality and morbidity. Restricted therapeutic choices are available to improve neurological impairment and disability in these patients. ACP treatment is safe and is being increasingly applied in clinical practice worldwide. Early ACP shows potential benefits for these patients, especially within 7 days after stroke. Subgroup analysis suggested a potential trend where ACP may offer more benefits to the AD repair group. Larger prospective studies with control groups should be carried out in the future to draw convincing conclusions.

## Data Availability

The data that support the findings of this study are available from Chung Gung Memorial Hospital but restrictions apply to the availability of these data, which were used under license for the current study, and so are not publicly available. Data are however available from the authors upon reasonable request and with permission of Chung Gung Memorial Hospital.

## Abbreviations

ACP: Acupuncture
AD: Aortic dissection
ATAAD: Acute type A aortic dissection
BI: Barthel Index
BMI: Body mass index
CABG: Coronary artery bypass grafting
CT: Computed tomography
GCS: Glasgow Coma Scale
IQR: Interquartile range
MD: Median difference
MRI: Magnetic resonance imaging
MS: Muscle strength grading scale
SD: Standard deviation
